# Structure-Templated Predictions of Novel Protein Interactions from Sequence Information

**DOI:** 10.1371/journal.pcbi.0030182

**Published:** 2007-09-21

**Authors:** Doron Betel, Kevin E Breitkreuz, Ruth Isserlin, Danielle Dewar-Darch, Mike Tyers, Christopher W. V Hogue

**Affiliations:** 1 Samuel Lunenfeld Research Institute, Mt. Sinai Hospital, Toronto, Ontario, Canada; 2 Department of Biochemistry, University of Toronto, Toronto, Ontario, Canada; 3 Department of Medical Genetics and Microbiology, University of Toronto, Toronto, Ontario, Canada; Peking University, China

## Abstract

The multitude of functions performed in the cell are largely controlled by a set of carefully orchestrated protein interactions often facilitated by specific binding of conserved domains in the interacting proteins. Interacting domains commonly exhibit distinct binding specificity to short and conserved recognition peptides called binding profiles. Although many conserved domains are known in nature, only a few have well-characterized binding profiles. Here, we describe a novel predictive method known as domain–motif interactions from structural topology (D-MIST) for elucidating the binding profiles of interacting domains. A set of domains and their corresponding binding profiles were derived from extant protein structures and protein interaction data and then used to predict novel protein interactions in yeast. A number of the predicted interactions were verified experimentally, including new interactions of the mitotic exit network, RNA polymerases, nucleotide metabolism enzymes, and the chaperone complex. These results demonstrate that new protein interactions can be predicted exclusively from sequence information.

## Author Summary

Many functions performed within a living cell are mediated by specific interactions between proteins. Precise geometric and chemical matches between segments of the protein structures facilitate those interactions. Such binding surfaces are often evolutionarily conserved elements of protein structures known as conserved domains that recognize specific binding elements on the interacting proteins. Binding domains and their corresponding interacting profiles constitute basic interacting modules that are replicated in multiple protein pairs, where they mediate similar interactions. Although many conserved domains are identified, only a handful have known, well-characterized binding elements. This paper describes a computational method that aims to elucidate the binding specificity of many domains. The utility of the derived binding specificity is demonstrated by predicting new interactions between yeast proteins. The predictions are based solely on sequence information by identifying the conserved domains and their corresponding binding sequences. A number of the predicted interactions were confirmed experimentally, demonstrating the feasibility of this approach.

## Introduction

The interaction between two proteins is a geometric and electrostatic match between two polypeptide surfaces that results in a stable set of bonds between amino acid side chains or backbone atoms. The interacting amino acids are often part of conserved sequence features such as domains or short linear motifs that constitute the interaction site between the two proteins. Despite the increased coverage and sensitivity of experimental techniques for detecting protein interactions [1–6] (reviewed in [7]), elucidating the precise interacting residues remains experimentally difficult. In most cases, all that is known about an interaction is the identity of the two interacting proteins, with little information about the underlying binding site. However, detailed knowledge of interaction specificity is important for understanding reaction mechanism, interaction prediction, and drug development.

Interacting domains are autonomous structural elements that exhibit distinct binding specificity to a multitude of target polypeptides. Such domains act as independent elements that can be “plugged” into a new protein and thereby introduce new functionality to the emerging protein [8]. From an evolutionary perspective, such rearrangements and the multiplication of existing conserved domains is a likely mechanism by which organisms generate new proteins, pathways, and novel functionalities [9,10]. Several protein interaction prediction methods exploit the conservation of protein-binding interfaces by identifying domain pairs that consistently co-occur in interacting proteins or coevolve, which are then used to predict new interactions [11–16]. Structure-based prediction methods use known protein complexes to model interactions between proteins that are homologous to the complex components [17,18]. Other prediction methods use integrative approaches that incorporate interaction experiments with additional functional information such as correlated expression level, common functional annotation [19,20], and cross-species comparisons [21]. Alternative approaches attempt to identify correlated sequence motifs that represent generic interacting sequence elements that may or may not be components of conserved domains [22–25]. In a few limited cases, detailed experimental data are used to generate high-resolution definition of domain binding profiles; however, such information is available only for a small number of domains [26,27].

**Figure 1:**
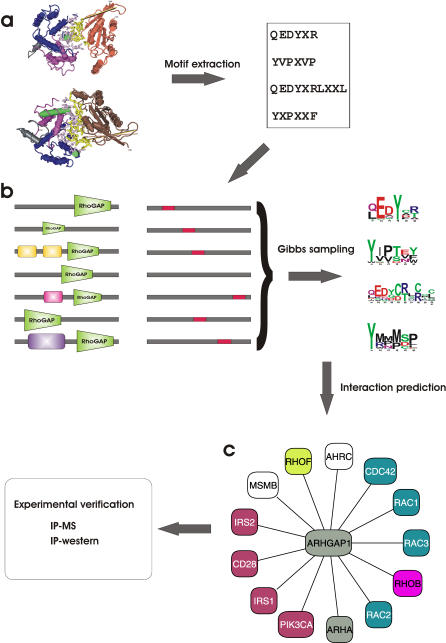
Outline of D-MIST Method for Predicting Protein Interactions by Learned Binding Profiles Identification of domain-binding profiles begins by extracting the short sequence motifs from structural complexes that contain the domain of interest. (A) In this example, RhoGAP-interacting motifs are extracted from two structural complexes (PDB ID 1AM4, 1TX4) where RhoGAP is bound to small G proteins. (B) Protein interactions containing the RhoGAP domain were collected from four databases to form the learning set for the Gibbs sampling to generate the binding profiles (shown here as sequence logos [57]). The sampling step is biased towards motifs that are similar to those found in the structural dataset. (C) The resulting PSSMs are used to predict interactions for proteins with RhoGAP domains, such as the human ARHGAP1. A subset of the predicted interactions is subsequently tested by two experimental methods.

Our primary objective is to predict interaction between proteins strictly from sequence information. Our approach is based on identifying the binding specificity of interacting domains that can then be used to predict new interactions. Here, we use existing physical interaction data to derive sequence profiles of the binding sequences that are presumed to determine the binding specificity of interacting domains. Our method, called domain–motif interactions from structural topology (D-MIST), is based on a two-step approach. First, potential domain-binding motifs are extracted from structural data. Second, these motifs are converted to sequence profiles in the form of position-specific scoring matrices (PSSMs). These PSSMs are derived using a subset of experimentally determined binary interactions that contain the domain of interest (Figure 1). Gibbs sampling, seeded with the motif extracted from structural data, is used to generate a PSSM from similar sequences that occur in a subset of established interacting proteins. We used the domain-binding profiles to predict protein interactions in yeast. The predictions were compared to a hidden set of known interactions reported in the literature, and several predicted interactions were confirmed directly by in vivo coprecipitation experiments.

## Results

The library of 3-D structures of protein complexes contains a detailed description of the binding interfaces between interacting proteins that include atom contacts and residue side-chain interactions [28]. Using more than 10,000 structural complexes, we identified the domains in the binding sites and extracted their associated sequence motifs on the opposing chain. Interacting residues were defined as two residues on opposite polypeptide chains separated by a maximum of 5 Å (Figure 1A). On average, each domain had two spatially separated interacting sequence motifs per interaction. Most domains were present in multiple 3-D structures in a variety of conformations, resulting in varied interacting sequence motifs with different levels of similarities.

The binding specificity of a domain is determined by a combination of physiochemical properties and structural constraints at the binding site that can be satisfied by multiple variations of the consensus sequence motif [29]. The interacting sequence motifs extracted from the protein structures represent a first approximation of the binding specificity of the interacting domains, but do not represent the full evolutionary variations of the residue–residue interactions available in one binding topology. A more informative representation of the possible motif variations is a sequence profile in the form of a PSSM that captures the compositional variance by assigning probabilities to each amino acid at each position. These sequence variations of the binding profiles can be learned from proteins that are known to interact through the same domain.

We collected a set of 87,894 nonredundant protein interactions from four databases containing binary protein interactions from multiple species. Interactions derived from structural studies were excluded to preclude self-identification, as well as high-throughput protein complexes identification experiments [30,31] (see Methods). Gibbs sampling [32] was used to learn the PSSM binding profiles for a specific domain by sampling positions in the set of proteins that interact with proteins that contain the domain of interest. The majority of the proteins in the learning set are assumed to interact through the common domain, and the generated PSSM will represent its binding profile (Figure 1B). Gibbs sampling enables the incorporation of prior knowledge about the length and composition of the binding profiles. The motifs identified in the 3-D structural analysis were used as prior knowledge in seeding the profile detection step to bias the sampling towards similar sequence regions. The result is a set of sequence PSSMs that represent the binding profiles of the interacting domains (Text S1).

The learned PSSMs were used to predict interactions for 703 yeast proteins with domains for which we successfully derived binding profiles. A physical interaction was predicted between proteins containing interacting domains and proteins with one or more of the interacting profiles associated with those domains (Figure 1C). A total of 18,459 interactions were predicted between 2,313 proteins (Dataset S1). We compared the predicted interactions to a comprehensive list of physical and genetic yeast interactions extracted from the literature [33] and found that 609 predicted interactions have reported experimental evidence (∼3%; *p =* 1.0 × 10^−13^; Figure S1). We note that 591 predicted interactions were found in both the 87,894 set of interactions used for the PSSM derivation and in the set of yeast literature curated interactions (∼32,000). However, none of the 609 predicted interactions that have supporting evidence in the literature overlap with those common 591 interactions. We did not incorporate additional experimental information such as cellular localization, functional annotation, surface accessibility, or gene expression data that would likely improve our prediction accuracy given that our primary goal was to predict novel interactions exclusively from sequence information.

**Figure 2:**
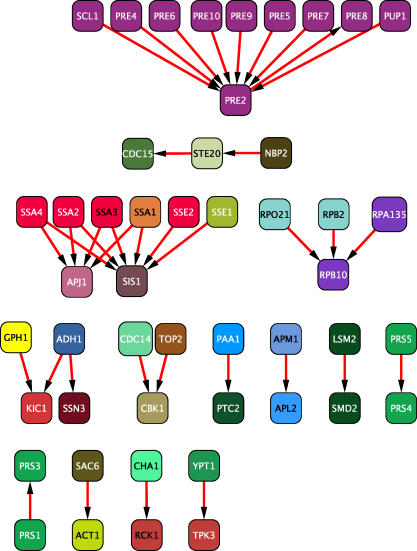
Predicted Interactions Verified by IP-MS Immunoaffinity purification of bait proteins complexes followed by mass spectrometry identification of associated proteins confirmed 37 predicted interactions. Predictions between proteins that were both co-purified with the tagged bait protein (i.e., both proteins were prey) were not considered validated. Proteins are coloured according to their Gene Ontology biological process annotation.

**Figure 3:**
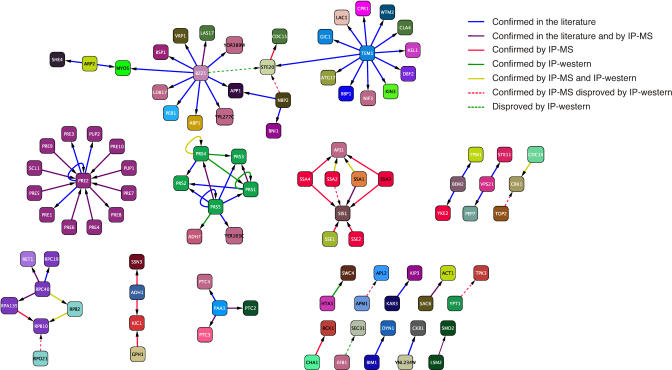
Predicted Interactions Confirmed by Experiments or by Previously Published Results in the Primary Literature Interactions are coloured according to their verification source. Dashed red lines are predictions that were confirmed by IP-MS but not confirmed by IP-western; dashed green lines are predictions that failed experimental validation by IP-western.

Experimental verification of a subset of the predicted interactions was performed by a one-step immunoaffinity purification of one of the two interaction partners, followed by mass spectrometric identification of associated proteins (IP-MS) as previously described [31]. The IP-MS method confirmed 37 predicted interactions, including 23 novel interactions (Figure 2). As a second means to experimentally verify our predictions, we immunoprecipitated one protein in the interacting pair, followed by antibody detection of the second protein (IP-western), also as described in [31]. The IP-western method reaffirmed five of the interactions confirmed by IP-MS (yellow edges; Figure 3) and identified an additional four novel interactions (green edges; Figure 3). We note that six interactions confirmed by the IP-MS approach were not detected by IP-western (red dashed edges; Figure 3); this discrepancy may be due either to nonspecific interactions detected by IP-MS or to interference of the second epitope tag with some interactions and/or expression levels in vivo. Of the 18 predictions that were tested by IP-western, nine novel interactions were confirmed, and a total of 30 new interactions were identified by both the IP-MS and IP-western methods.

### Experimentally Confirmed Predictions

Among the experimentally confirmed predictions were interactions between the five components of the PRS complex, which together compose the 5-phosphoribosyl-1(a)-pyrophosphate synthetase enzyme (EC number 2.7.6.1). This complex is a key component in the production of the precursors for purine, pyrmidine, and pyridine nucleotides [34]. An additional interaction was confirmed between the alcohol dehydrogenase (NADP^+^) Adh7 and Prs5, the latter being a member of the PRS complex. This result suggests a possible direct link between NADP/NADPH balance, which is controlled by Adh7 [35], and the biosynthesis of the purine and pyrimidine precursors. A predicted interaction between the histone H2A protein Hta1 and God1, a component of the SWR-C protein complex that incorporates Htz1 into the chromatin, was also confirmed. Chromatin remodelling by the exchange of Hta1 with Htz1 is thought to induce chromatin restructuring that favours gene transcription, RNA polymerase II recruitment, and gene expression induction near silent heterochromatin [36]. Another confirmed interaction is between a member of the HSP40 family (Apj1) with two HSP70 proteins (Ssa1, Ssa2). HSP40 family members form complexes with HSP70 chaperone proteins, which facilitate the folding of specific proteins at various cellular locations [37]. We also identified new interactions between the RNA polymerase II subunit Rpb2 with Rpb10, which is a common subunit of all three RNA polymerases [38]. Additional interaction was demonstrated between Rpc40, a known shared subunit of RNA polymerases I and III, and Rpb2, an exclusive component of RNA polymerase II. It is possible that some of these interactions are bridged or stabilized by other RNA polymerase subunits [39].

One might argue that the above successful predictions could be easily predicted from the orthology of the interacting proteins to the structural complexes used, such as the interactions between members of the PRS complex. We therefore tested several nonobvious predicted interactions that cannot be easily inferred from structural or sequence homology to other interacting pairs. The critical downstream effector of the mitotic exit network is the phosphatase Cdc14, which activates Clb degradation and Sic1 accumulation by dephosphorylation of key substrates [40]. We confirmed an unexpected predicted interaction between Cdc14 and the protein kinase Cbk1, which functions in a parallel pathway (called RAM [regulation of Ace2p activity and cellular morphogenesis]) at the end of mitosis to facilitate cytokinesis and mother–daughter abscission [41]. The Cdc14–Cbk1 interaction suggests that the activity of the mitotic exit network and RAM pathways may be coordinated via Cdc14-mediated dephosphorylation of RAM components and/or Cbk1-mediated phosphorylation of mitotic exit network components [42]. Other nonobvious interactions between known components of clathrin-associated (AP-1) complex Apm1 and Apl2, as well as between components of the RNA splicing complex Smd2 and Lsm2, were detected by the IP-MS experiments but not by IP-western under the conditions used. Given the strength of the D-MIST predictions for these latter interactions, further investigation using more sensitive reagents seems warranted. These confirmed predictions of nonobvious interactions illustrate the potential of the D-MIST approach to generate new biological hypotheses.

## Discussion

As noted previously, we excluded additional experimental evidence, such as localization and expression data from our prediction method. Although additional experimental information and functional annotation would likely improve prediction accuracy, it may also limit predictions only to those proteins with prior experimental or functional information. In addition, the use of functional annotation such as Gene Ontology terms (assigned by human experts or predicted computationally) in a prediction method will penalize predicted interactions between proteins with unrelated functions. Therefore, it restricts the ability to predict interactions between apparently unrelated proteins that could illuminate new cellular functions [43].

The D-MIST method for identifying domain-binding modules is currently limited in a number of ways. The first limitation is the availability of detailed binding information, as attained primarily through structural studies and peptide-based approaches such as phage display [44] and random peptide libraries [45]. In addition, several studies have concluded that the repertoire of protein structures in the Protein Data Bank is significantly biased in that *trans*-membrane and disordered domains are underrepresented due to limitations in structure determination [46,47]. Consequently, D-MIST analysis that depends on structural representation of protein interactions is similarly biased. The existing detailed examples of interactions are therefore sparse and noncomprehensive, with only a small subset of all possible domains that is represented. The second limitation is that the derived motifs do not represent the entire repertoire of all possible domain-binding sequences, even for those domains where structural data exist. The third limitation arises from the statistical framework of the Gibbs sampling method that requires a sufficient number of proteins to sample from in order to converge towards a meaningful PSSM. We restricted the analysis to domains with five or more putative interactors, thereby excluding domains that are infrequently found in our set of protein interactions. Fourth, some domains are not amendable to this type of analysis due to the diverse nature of their binding motifs that lack sequence conservation [29]. Last, many interactions are governed by posttranslational modifications or precise physiological states, which may also hamper the accuracy of D-MIST predictions. Despite the above limitations, we have shown that novel protein interactions can be predicted strictly from primary sequence information. D-MIST not only predicts interactions between proteins but also provides sequence level predictions about the binding sites that can be verified experimentally. Predicting protein interactions without the need for additional information or prior experiments is particularly valuable when studying uncharacterized proteins and for predicting interactions in poorly studied organisms where typically only sequence information and predicted open reading frames are available. The sole dependence on sequence information allows for interaction prediction in other organisms without further modifications to the method or input datasets. With the advent of structural genomics initiatives [48], the power of the D-MIST approach will certainly increase.

## Methods

### Extracting motifs.

The domain-binding motifs were extracted from BIND protein interaction records that were generated from 10,064 structures [28]. Interactions were filtered for crystal-packing artifacts using the PQS server [49], and all the interactions are available as a subset of the BIND database. Domain annotation was assigned to the protein structures using our in-house adaptation of CDD [50] with an e-value cutoff of 10 × 10^−6^ and then converted to InterPro identifiers [51]. Binding motifs are defined as polypeptide segments of five residues or longer in which the amino acids side chains are <5 Å from the interacting domain's side chains on the opposing protein. Two motif residues that are in direct contact with the interacting domain can be separated by a maximum of two noncontacting residues. For example, the first motifs in Figure 1A contain a tyrosine and an arginine that are within 5 Å from the side chains of the RhoGAP domain separated by a distal residue, marked by X, that is not within contact range with the RhoGAP domain.

### Learning the binding modules.

A total of 87,894 nonredundant protein interactions were collected from 204 species from four database sources: BIND [52], DIP [53], Mint [54], and IntAct [55]. We excluded all interactions that were derived from 3-D studies, high-throughput protein complex identification studies [30,31], or interactions inferred from synthetic lethal experiments. The interactions were indexed in a relational database by domain annotation such that a single query can provide the full list of proteins that interact with a domain of interest (Figure 1B). We used Gibbs sampling [32] seeded with sequence motifs identified in the structural studies to compute a PSSM using the subset of pairwise protein interactions that contain the domain to which the motif was bound in the 3-D structure. The length of the structural motifs was used to approximate the length of the PSSMs. The frequency of residue *j* at position *i* in the PSSM (the *i,j* entry in the matrix) is computed as follows:

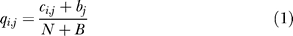
where *c_i,j_* is the observed counts of residue *j* at position *i* in the sampled proteins, *b_j_* is pseudocounts for residue *j*, *N* is the number of sequences sampled, and *B* is the total number of pseudocounts for all residues. By increasing the pseudocount term (*b_j_*) for specific positions in the PSSM, the sampling algorithm is biased to favour positions where the residue at position *i* in the sampled protein is similar to the residue at position *i* of the structural motif. We set the pseudocounts to equal 62% of the residue counts in the sampled proteins.

### Predicting new interactions.

Two proteins were predicted to interact if one protein had a domain and a second protein matched one or more of the binding profiles for that domain (Figure 1C). We attempted to predict interactions between all yeast proteins by searching for domain-binding profiles as described in [56] using PSSMs with a score cutoff >10.0 (as scored by the Gibbs sampler) and a cutoff >0.20 for the match between the PSSM and the protein. Potential interactors among the yeast proteome were identified for 703 domain-containing proteins with derived binding profiles. In total, 18,459 interactions were predicted between 2,313 proteins based on the presence of a domain and its binding profile in the interacting pair.

### Experimental verification.

Recombination-based cloning, culture growth, and protein complex isolation were performed essentially as described [31] with minor modifications. Each uncharacterized open reading frame was tagged at the 3′-end with the FLAG-tag epitope using the Gateway recombination-based cloning system (Invitrogen, http://www.invitrogen.com). Bait complexes were immunopurified on anti-FLAG M2 antibody resin, resolved by denaturing gel electrophoresis, and visualized by colloidal Coomassie stain. Protein identification by automated liquid chromatography tandem mass spectrometry on a Finnigan LCQ DECA ion trap (Thermo Finnigan, http://www.thermo.com) mass spectrometer was as described previously [31]. Predicted protein interactions were also confirmed by IP-western [31] using interaction partners tagged either as C-terminal HA or Myc_3_ epitope fusions and detection with 12CA5 anti-HA or 9E10 anti-Myc monoclonal antibodies, respectively (Figure S2).

### Overlap with literature.

The predicted interactions were compared to a new set of yeast curated interactions collected from more than 50,000 abstracts and publications [33] (available at www.thebiogrid.org). The probability of the observed overlap between the predicted interactions and the literature curated is approximated by a Poisson distribution. A random variable *Y* has a Poisson distribution if

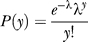
where *λ* = *Np*, *N* is the sample size, and *p* is the probability of a single event; i.e., the probability of selecting a true interaction by random chance. In the current analysis, *N* is the number of predicted interactions (18,459), *y* is the number of literature-validated predictions (609), and *p* is the probability of predicting a correct interaction by random chance for the 703 proteins for which interactions were predicted. The value of *p* is approximated as the frequency of true interactions among all possible protein pairs that were considered. Since there is no known complete set of interactions for any reference organism, we cautiously assume an upper bound of 100 physiological interactions per bait protein. This number is likely an order of magnitude larger than the true value. Potential interactors for the 703 proteins containing domains with derived binding profiles were identified by scanning the entire yeast proteome (∼6,000 proteins) for proteins that matched the domains binding profiles. Hence, the total number of proteins pairs that were considered (i.e., the entire search space) is 703 × 6,000. The value of *p* is then 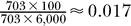 . Given these parameters *P* (*y* ≥ 609) under a Poisson distribution is 1.0 × 10^−13^. Similar calculation using a hypergeometric distribution (sampling without replacement) yields a *p*-value of 1.0 × 10^−8^.

## Supporting Information

Dataset S1 Cytoscape Session File Containing the Validated and Predicted Protein Interactions A Cytoscape session file containing the complete set of predicted interactions as well as the networks in Figures 2, 3, S1, and S2. The networks can be viewed using the Cytoscape network visualization tool freely available at http://www.cytoscape.org.
(2.0 MB ZIP)

Figure S1 The Overlap between the Predicted Interaction Network and a Comprehensive Set of Literature-Curated Interactions [33] The predicted interactions were compared to a new and exhaustive set of curated interactions extracted from the literature that includes physical interactions from both high-throughput and directed studies as well as genetic interactions. The overlap contains 609 interactions that represent ∼3% of the predicted interactions. Proteins are coloured according to Gene Ontology biological process annotation.
(519 KB PDF)

Figure S2 IP-Western Results for the Novel Interactions Predicted by D-MIST Bait proteins were purified using FLAG antibodies, and their interacting proteins were detected by antibodies specific to C-terminal HA or Myc3 epitopes.
(325 KB PDF)

Text S1 The Domain-Binding Profiles Derived by D-MIST Each domain-binding profile is specified as a list of sequence motifs. The sequence motifs are used as input to a PSSM search program [56]. Source code available at http://www.people.fas.harvard.edu/~junliu/index1.html#Computational_Biology.
(2.7 MB TXT)

## Abbreviations

D-MIST: domain–motif interactions from structural topology
IP-MS: immunoprecipitation followed by mass spectrometry
IP-western: immunoprecipitation followed by Western blotting
PSSM: position-specific scoring matrix
