# Supplementary material for: Structure-Templated Predictions of Novel Protein Interactions from Sequence Information
Source: PLoS Comput Biol. 2007 Sep 21;3(9):e182. doi: 10.1371/journal.pcbi.0030182 (PMC1988853; doi:10.1371/journal.pcbi.0030182)

# Supplementary Figure S1

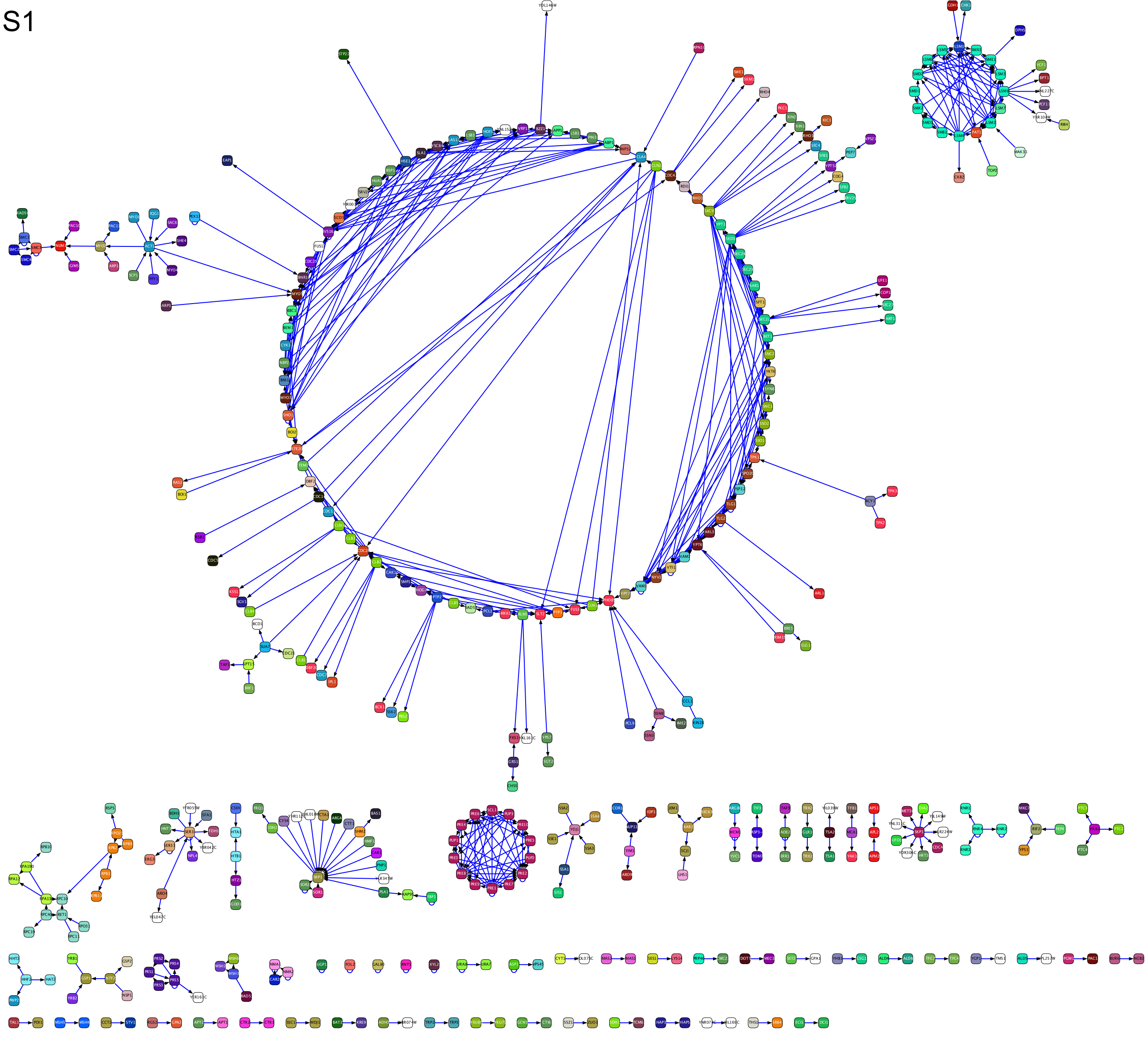

Supplement: Supplementary file 1 [file pcbi.0030182.sg001.pdf]
