# Supplementary material for: Structure-Templated Predictions of Novel Protein Interactions from Sequence Information
Source: PLoS Comput Biol. 2007 Sep 21;3(9):e182. doi: 10.1371/journal.pcbi.0030182 (PMC1988853; doi:10.1371/journal.pcbi.0030182)

Supplementary Figure S2

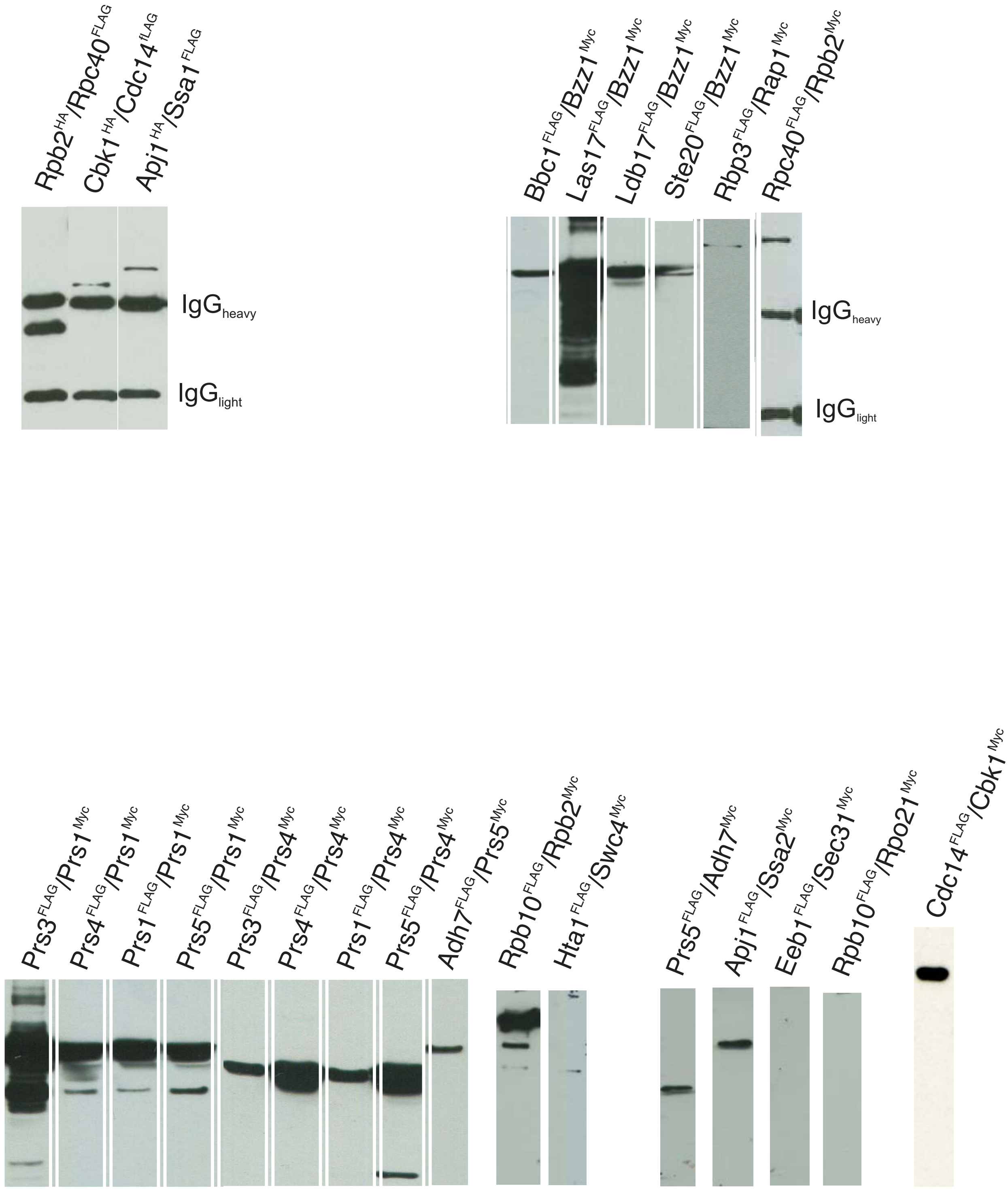

Supplement: Supplementary file 2 [file pcbi.0030182.sg002.pdf]
